# Impact of shifting from laparoscopic to robotic surgery during 600 minimally invasive pancreatic and liver resections

**DOI:** 10.1007/s00464-022-09735-4

**Published:** 2022-11-18

**Authors:** Anouk. M. L. H. Emmen, B. Görgec, M. J. W. Zwart, F. Daams, J. Erdmann, S. Festen, D. J. Gouma, T. M. van Gulik, J. van Hilst, G. Kazemier, S. Lof, S. I. Sussenbach, P. J. Tanis, B. M. Zonderhuis, O. R. Busch, R. J. Swijnenburg, M. G. Besselink, for HPB-Amsterdam

**Affiliations:** 1 Department of Surgery, Amsterdam UMC, University of Amsterdam, Amsterdam, The Netherlands; 2 Cancer Center Amsterdam, Amsterdam, The Netherlands; 3 Department of Surgery, Amsterdam UMC, Vrije Universiteit, Amsterdam, The Netherlands; 4 Department of Surgery, OLVG, Amsterdam, The Netherlands

**Keywords:** Minimally invasive, Pancreatoduodenectomy, Distal pancreatectomy, Liver surgery

## Abstract

**Background:**

Many centers worldwide are shifting from laparoscopic to robotic minimally invasive hepato-pancreato-biliary resections (MIS-HPB) but large single center series assessing this process are lacking. We hypothesized that the introduction of robot-assisted surgery was safe and feasible in a high-volume center.

**Methods:**

Single center, post-hoc assessment of prospectively collected data including all consecutive MIS-HPB resections (January 2010–February 2022). As of December 2018, all MIS pancreatoduodenectomy and liver resections were robot-assisted. All surgeons had participated in dedicated training programs for laparoscopic and robotic MIS-HPB. Primary outcomes were in-hospital/30-day mortality and Clavien-Dindo ≥ 3 complications.

**Results:**

Among 1875 pancreatic and liver resections, 600 (32%) were MIS-HPB resections. The overall rate of conversion was 4.3%, Clavien-Dindo ≥ 3 complications 25.7%, and in-hospital/30-day mortality 1.8% (*n* = 11). When comparing the period before and after the introduction of robotic MIS-HPB (Dec 2018), the overall use of MIS-HPB increased from 25.3 to 43.8% (*P* < 0.001) and blood loss decreased from 250 ml [IQR 100–500] to 150 ml [IQR 50–300] (*P* < 0.001). The 291 MIS pancreatic resections included 163 MIS pancreatoduodenectomies (52 laparoscopic, 111 robotic) with 4.3% conversion rate. The implementation of robotic pancreatoduodenectomy was associated with reduced operation time (450 vs 361 min; *P* < 0.001), reduced blood loss (350 vs 200 ml; *P* < 0.001), and a decreased rate of delayed gastric emptying (28.8% vs 9.9%; *P* = 0.009). The 309 MIS liver resections included 198 laparoscopic and 111 robotic procedures with a 3.6% conversion rate. The implementation of robotic liver resection was associated with less overall complications (24.7% vs 10.8%; *P* = 0.003) and shorter hospital stay (4 vs 3 days; *P* < 0.001).

**Conclusion:**

The introduction of robotic surgery was associated with greater implementation of MIS-HPB in up to nearly half of all pancreatic and liver resections. Although mortality and major morbidity were not affected, robotic surgery was associated with improvements in some selected outcomes. Ultimately, randomized studies and high-quality registries should determine its added value.

**Graphical Abstract:**

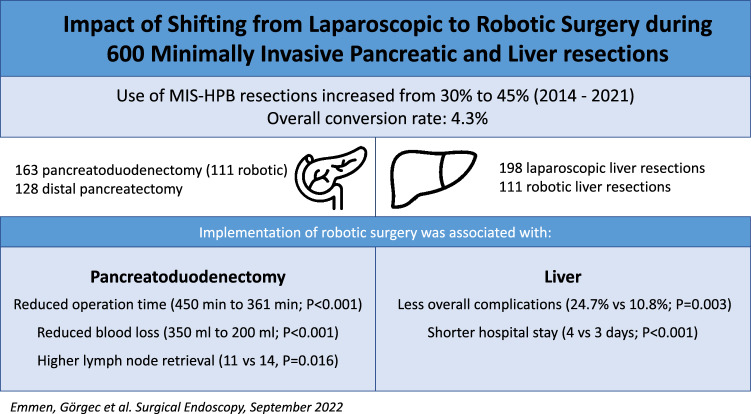

**Supplementary Information:**

The online version contains supplementary material available at 10.1007/s00464-022-09735-4.

Minimally invasive surgery (MIS) has become routine practice in most types of gastrointestinal surgery. After a slow adoption in hepato-pancreato-biliary (HPB) surgery in the 1990s and 2000s [1], MIS-HPB has seen a rapid development in high-volume centers. Over the last decade the use of robotic surgery is increasing rapidly.

For MIS distal pancreatectomy, two randomized trials reported faster functional recovery, shorter hospital stay, and less blood loss after the laparoscopic as compared to the open approach [2, 3]. Therefore, the recent Miami guidelines now advise MIS distal pancreatectomy for benign and low-grade malignant tumors. For laparoscopic pancreatoduodenectomy, four randomized trials showed shorter hospital stay although safety concerns were reported in one multicenter randomized trial [3–6]. The Miami guidelines advice a minimum annual center volume of 20 MIS pancreatoduodenectomies [7]. Good outcomes were reported from (mostly single) high volume centers for robotic pancreatoduodenectomy but randomized trials are lacking [8–11].

For MIS liver surgery, two randomized trials reported less complications and shorter hospital stay after laparoscopic resection of colorectal liver metastases [12, 13]. The Southampton consensus guidelines recommend laparoscopy as standard of care for minor liver resections [14]. Again, good outcomes were reported from (mostly single) high volume centers for robotic MIS liver surgery but randomized studies are lacking [15–20].

However, MIS-HPB resections are technically demanding and associated with long learning curves [21, 22] requiring structured training programs [7, 14] which were implemented in the Netherlands for MIS pancreas resections [8, 23, 24] and liver resections [25]. Robotic surgery may facilitate surgeons when performing the relatively challenging MIS-HPB resections but studies focusing on a HPB unit perspective (i.e., including both pancreatic and liver resections) are lacking. We hypothesize that the introduction of robot-assisted surgery might be safe and feasible and might enhance the overall implementation of MIS-HPB resections.

## Materials and methods

This is a single center study with post-hoc assessment of prospectively maintained database of all MIS-HPB resections, both laparoscopic and robot-assisted (hereafter: robotic) surgery between January 2010 and February 2022 at Amsterdam UMC. All consecutive procedures were included, including the first procedures for each type of resection. Most procedures were performed at the AMC hospital of Amsterdam UMC (January 2010 – May 2021). In June 2021, the AMC team moved to and merged with the HPB team in the VUMC hospital of Amsterdam UMC and the first 8 months hereafter were also included (June 2021 – February 2022). Inclusion ended when 600 MIS-HPB resections (i.e., including conversions) had been performed. All data were collected from the mandatory and anonymized, prospective Dutch Pancreatic Cancer Audit (DPCA) and Dutch Hepatobiliary Audit (DHBA) registries. The study was reported in accordance with the Strengthening the Reporting of Observational Studies in Epidemiology (STROBE) statement [26]. The ethical committee of the Amsterdam UMC decided that ethical approval was not needed for this study since participants were not subject to additional interventions. Hence, no written informed consent was collected.

### Eligibility criteria

Included were all MIS-HPB resections for which indications did not change during the study period. For MIS pancreatic resections this included laparoscopic and robotic pancreatoduodenectomy and distal pancreatectomy. Indications for MIS pancreatoduodenectomy included tumors < 8 cm, absence of portomesenteric vein or arterial involvement (3-5 mm free margin) on a computed tomography of maximum 4 weeks old, absence of chronic pancreatitis, and a BMI < 35 kg/m^2^. Indications for MIS distal pancreatectomy included tumors < 8 cm, absence of portomesenteric vein and celiac trunc involvement (3–5 mm free margin) on a computed tomography of maximum 4 weeks old.

MIS liver resections included both minor and major laparoscopic and robotic liver resections. Indications for MIS liver resections include tumors < 10 cm, absence of portal vein involvement (5-10 mm free margin) in the first 50 procedures, absence of caval vein involvement, and absence of need for a hilar resection. Previous abdominal surgery was not a contra-indication for MIS-HPB surgery. All MIS-HPB drainage surgery (such as for acute pancreatitis, chronic pancreatitis, liver cysts) and less common resections such as MIS central pancreatic and MIS total pancreatectomy were excluded from the current analysis. Patient undergoing conversion were included according to the intention-to-treat principle.

### Surgical techniques

The techniques of laparoscopic and robotic MIS pancreatoduodenectomy and MIS distal pancreatectomy have previously been described in the LAELAPS-1, -2, and -3 training programs [8, 23, 24]. The da Vinci® Xi Robotic Surgical System (Intuitive Surgical®, Inc., Sunnyvale, CA, USA) was used since December 2018 for all MIS pancreatoduodenectomy procedures and essentially all MIS liver resections. In all robotic procedures, the table-side surgeon has a large standalone 3D-display on the patients’ right side. For laparoscopic liver resection, as previously described, a standardized approach was used [25]. For robotic liver resection, superficial parenchymal transection was performed by monopolar cautery and a vessel sealer. For deep parenchymal transection, a vessel sealer was used in combination with a robotic Maryland bipolar device. Indocyanine green (ICG) fluorescence imaging was administered perioperatively for different applications: 24 h prior to surgery for tumor imaging, within 1 h prior to surgery for biliary tract mapping, and intra-operatively for liver perfusion assessment. Vascular and biliary structures were divided between Hem-o-Lok clips (Weck Closure Systems, Research Triangle Park, USA) or a vessel sealer, or endoscopic stapling as required. Anesthetic management generally involved a restrictive intravenous fluid approach during liver transection combined with a low central venous pressure. Pringle was used on demand. Specimens were extracted in a plastic endoscopic bag via a widened trocar incision for small lesions or a Pfannenstiel incision for larger lesions or anatomically major resections.

### Training surgical team

For laparoscopic pancreatoduodenectomy, which started in 2014, the surgical team consisted of 2 (out of 3) staff surgeons who all participated also in the LAELAPS-2 and -3 training programs [8, 24]. Per December 2018, 4 staff surgeons participated in the LAELAPS-3 training program for robotic pancreatoduodenectomy. Again, the surgical team per procedure involved 2 (out of 4) staff surgeons with one surgeon (MGB) present during all procedures to minimize the learning curve effect. Surgeons switched roles between the resection- and reconstruction phase. For MIS distal pancreatectomy, the surgical team involved 1 (out of 2) staff surgeons who participated also in the LAELAPS-1 training program surgeons, along with a fellow or senior resident [23]. Laparoscopic liver resection, which started in 2010, was performed by 1 or 2 (out of 4) staff surgeons, who participated also in the LAELIVE training program, along with a fellow or senior resident [27]. Per December 2018, 3 staff surgeons started with robotic liver resection. The surgical team involved 1 or 2 (out of 3) staff surgeons, along with a fellow or senior resident [23]. One surgeon (RJS) was present during all robotic resections to minimize the learning curve.

### Data collection and definitions

Baseline patient characteristics included age, sex, body mass index (BMI, kg/m2), American Society of Anaesthesiologists (ASA) grade, cirrhosis, neoadjuvant chemotherapy, previous extrahepatic abdominal surgery, previous liver surgery and origin of the tumor. Tumor and procedure characteristics included histological diagnosis, number of lesions, size of the largest lesion, type of resection (minor, technically major, anatomically major resection [28]), and extent of resection.

Operative outcome included 30-day overall postoperative complications (defined according to the Clavien-Dindo classification; severe/major complications were defined as Clavien-Dindo grade III or higher) [29], 30-day readmission, 30-day reoperation, postoperative length of hospital stay and 30-day/in hospital mortality and, according to the International Study Group of Pancreatic Surgery (ISGPS) grade B/C definitions: pancreatic fistula (POPF) [30], delayed gastric emptying (DGE) [31], and post-pancreatectomy hemorrhage (PPH) [32]. Postoperative bile leakage grade B/C was defined according to the International Study Group of Liver Surgery [33].

Minor liver resection was classified as any resection from the anterolateral segments, *i.e.,* 2, 3, 4b, 5, and 6 [28]. Anatomically major liver resection included resection of three or more Couinaud’s segments [28]. Technically major liver resection was considered any resection from the posterosuperior segments, *i.e.,* 7, 8, 4a and 1 [28].

### Outcome measures

The primary outcomes were in-hospital/30-day mortality and severe/major morbidity (≥ Grade 3a) according to the Clavien-Dindo classification [29]. Secondary outcomes included operative time, length of stay, blood loss, conversion, and R0/R1 resection margin.

### Statistical analysis

Data were analyzed using IBM SPSS Statistics for Windows version 27.0 (IBM Corp., Orchard Road Armonk, New York, US). Student’s t, Mann Whitney U, Chi-square, or Fisher’s exact tests will be used as appropriate. Categorical data are presented as proportions, continuous data are presented as either mean and standard deviation or median and inter-quartile-range as appropriate.

The learning effects were assessed by determining the correlation between consecutive procedures number (binning of 10 procedures) and the learning curve outcomes as determined by Müller et al.: (1) Competency: decrease in operative time; (2) Proficiency: decrease in major morbidity and mortality; and (3) Mastery: increase in textbook outcome. When a significant correlation was found, a CUSUM analysis was used to assess the learning curve. The top of the CUSUM graph thus represented the total operative time expressed in standard deviations above average up to that case. When interpreting the CUSUM graph, ‘slope’ is the informative part, wherein an uphill slope indicates an outcome above average and a downhill slope indicates an outcome below average for that consecutive case number. The turning point of curvature indicate the transition from one phase to another and overcomes the specific learning curve. For liver procedures, the CUSUM analysis was adjusted for either minor-, technically major-, or anatomically major resection.

## Results

**Fig. 1:**
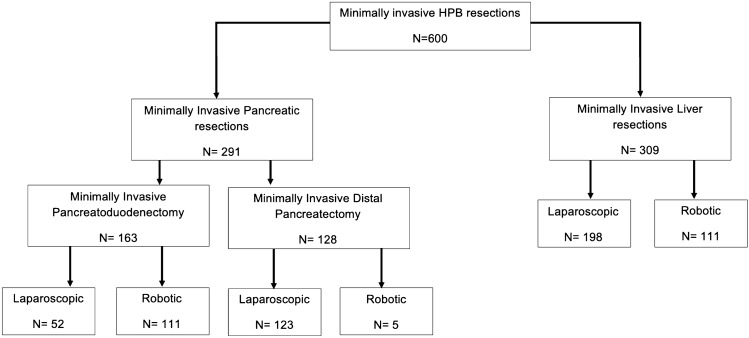
Flow diagram of all minimally invasive HPB procedures

During the study period, 1875 HPB resections were performed including 600 MIS-HPB procedures (32%) consisting of 291 MIS pancreatic, of which 163 pancreatoduodenectomy, and 309 MIS liver resections (Fig. 1).

### MIS-HPB patient characteristics and outcome

Table 1 shows the patient characteristics and outcome of all 600 patients undergoing a MIS-HPB resection. Median age was 64 years [IQR 53–72] with ASA 1–2 (73.6%). The median operation time was 281 min [IQR 171–374] with a median blood loss of 200 ml [IQR 100–400] and a conversion rate of 4.3%. Major morbidity occurred in 25.7% (*n* = 154) of patients. The median length of hospital stay was 5 days [IQR 3–8 days]. The overall in-hospital/30-day mortality was 1.8% (*n* = 11).

**Table 1:** Patient characteristics and outcomes of all minimally invasive pancreatic and liver resections

Characteristics	MIS-HPB resection *N* = 600*
Age, years, median [IQR]	64 (53–72)
Male, *n* (%)	326 (54.4)
*ASA score*	
ASA 1–2, *n* (%)	441 (73.6)
ASA 3–4, *n* (%)	158 (26.4)
BMI, kg/m2, median [IQR]	25.3 (22.7–28.6)
*Surgical approach*	
Laparoscopic (%)	373 (62.2)
Robotic (%)	227 (37.8)
Operation time, minutes, median [IQR]	281 (171–374)
Blood loss, ml, median [IQR]	200 (100–400)
Conversion *n* (%)	26 (4.3)
Clavien Dindo ≥ 3 (%)	154 (25.7)
Hospital stay, days, median [IQR]	5 (3–8)
Readmissio*n* < 30 days (%)	72 (13.4)
R0 resection in case of malignancy, *n* (%)	336 (86.6)
In-hospital mortality/30-day *n* (%)	11 (1.8)

Values in parentheses are percentages unless mentioned otherwise. Percentages may not add up due to rounding and missing data

*IQR* inter quartile range, *BMI* body mass index, *ASA* American Society of Anaesthesiology

*Including RDP (*n* = 5)

### Trends in time

**Fig. 2:**
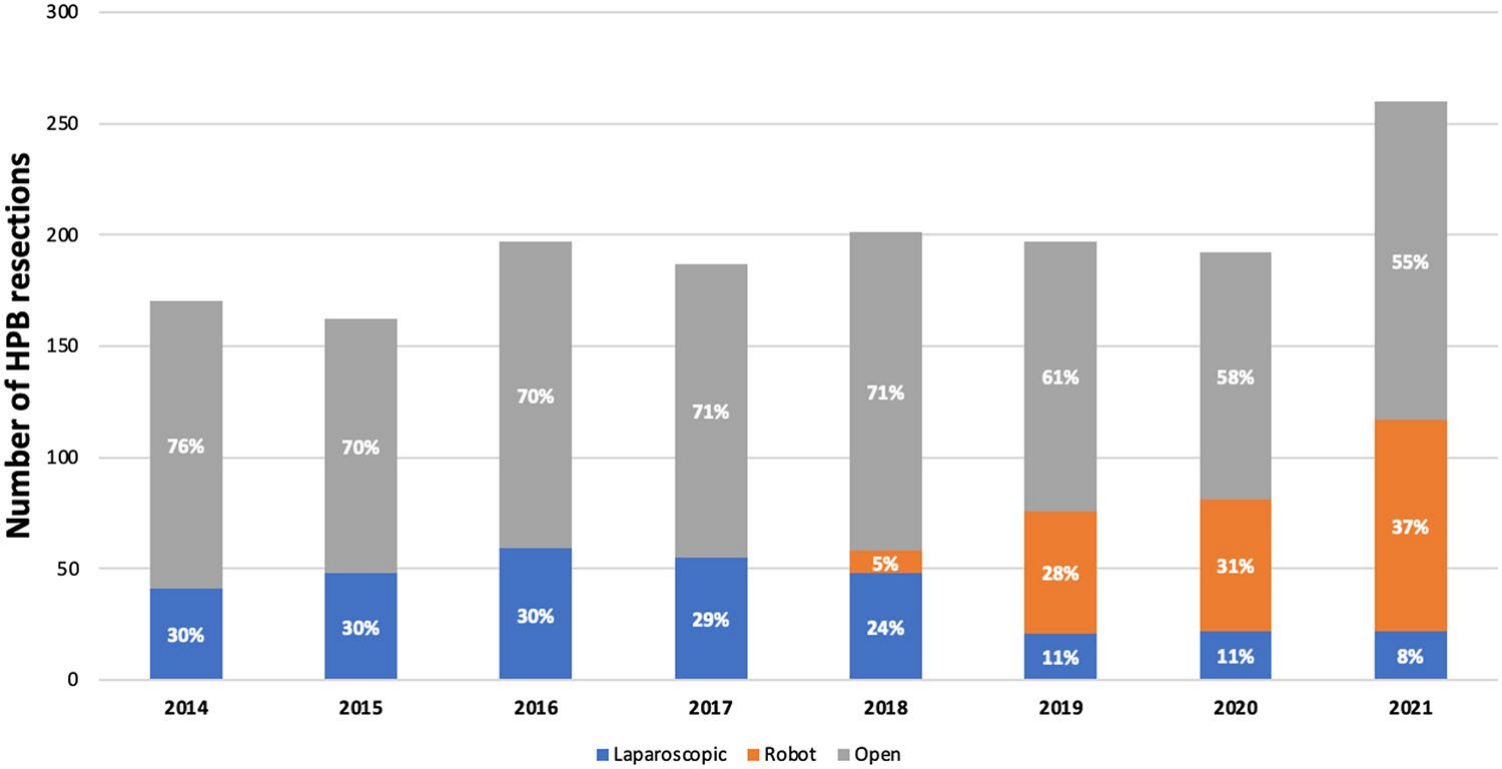
Total volume and annual rate of MIS-HPB in relation to all pancreatic and liver resections

Figure 2 shows the implementation rates of MIS-HPB per year. The overall use of MIS-HPB increased from 30% in 2014 to 45% in 2021 (*P* < 0.001). When comparing the period before and after the introduction of robotic MIS-HPB (Dec 2018), the overall use of MIS-HPB increased from 25.3% to 43.8% (*P* < 0.001). The median overall blood loss decreased from 250 ml [IQR 100–500] with laparoscopic MIS-HPB to 150 ml [IQR 50–300] with robotic MIS-HPB (*P* < 0.001).

### MIS pancreatectomy (all procedures)

Overall, 291 patients underwent MIS pancreatectomy during the study period including 175 laparoscopic and 116 robotic procedures. The implementation rate per year increased from 21.5% in 2014 to 44.2% in 2021 (*P* =  < 0.001), Supplementary Fig. 1. The vast majority of MIS distal pancreatectomy procedures (96.1%) was performed laparoscopically. The median annual volume of MIS pancreatectomy was 28 [IQR 23–49] which increased from 23 in 2014 to 68 in 2021 (*P* < 0.001).

### MIS pancreatoduodenectomy

Patient characteristics are shown in Table 2; 52 patients underwent laparoscopic and hereafter 111 patients robotic pancreatoduodenectomy. The median annual volume of MIS pancreatoduodenectomy was 25 [IQR 15–37], including 45 procedures in the last study year, 2021. The use of MIS pancreatoduodenectomy increased from 28.4% in 2015 to 36.3% of all pancreatoduodenectomies in 2021 (*P* = 0.253). Gland texture was most often soft (67.3% in laparoscopic and 63.1% in robotic).

**Table 2:** Patient, tumor, and procedural characteristics of all minimally invasive pancreatic resections

Characteristics	Laparoscopic pancreatoduodenectomy *N* = 52	Robotic pancreatoduodenectomy *N* = 111	P	Laparoscopic distal pancreatectomy *N* = 123
Age, years, median [IQR]	70 [58–78]	68 [61–74]	0.312	66 [54–73]
Male, *n* (%)	24 (46.2)	67 (60.4)	0.089	59 (48)
*ASA score*			0.081	
ASA 1–2. n (%)	42 (80.8)	75 (67.6)		90 (73.2)
ASA 3–4, n (%)	10 (19.2)	36 (32.4)		33 (26.8)
BMI, kg/m2, median [IQR]	24.2 [21.5–27.9]	24.9 [22.2–27.1]	0.481	26.3 [22.7–29.3]
Neo adjuvant therapy, n (%)	–	6 (5.4)	0.057	7 (5.7)
Size lesion median [IQR]	18 [15–30]	23 [17–32]	0.115	30 [21–50]
*Origin*			0.724	
Pancreas	21 (40.4)	50 (45)		115 (93.5)
Distal bile duct	10 (19.2)	23 (20.7)		1 (0.8)
Ampullary	18 (34.6)	25 (22.5)		–
Duodenum	2 (3.8)	10 (9.0)		–
Other	1 (1.9)	3 (2.7)		7 (5.7)
*Histological diagnosis*			0.357	
Adenocarcinoma	33 (63.5)	63 (56.8)		25 (20.3)
Intraductal papillary mucinous neoplasm	9 (17.3)	20 (18.0)		29 (23.6)
Mucinous cystic neoplasm (MCN)	–	1 (0.9)		7 (5.7)
Neuroendocrine tumor	5 (9.6)	10 (9.0)		28 (22.8)
Solid-pseudopapillary neoplasm	–	–		5 (4.1)
Adenoma	1 (1.9)	7 (6.3)		–
Pancreatitis	–	2 (1.8)		14 (11.4)
Other	4 (7.7)	8 (7.2)		15 (12.2)
*Lymph nodes resected in malignant*				
Disease, median [IQR]	11 [8–15]	14 [11–17]	0.016	11 [5–16]
Involved lymph nodes, median [IQR]	1 [0–3]	1 [0–3]	0.580	0 [0–2]

Values in parentheses are percentages unless mentioned otherwise. Percentages may not add up due to rounding and missing data

*IQR* interquartile range, *BMI* body mass index, *ASA* American Society of Anaesthesiology

**Fig. 3:**
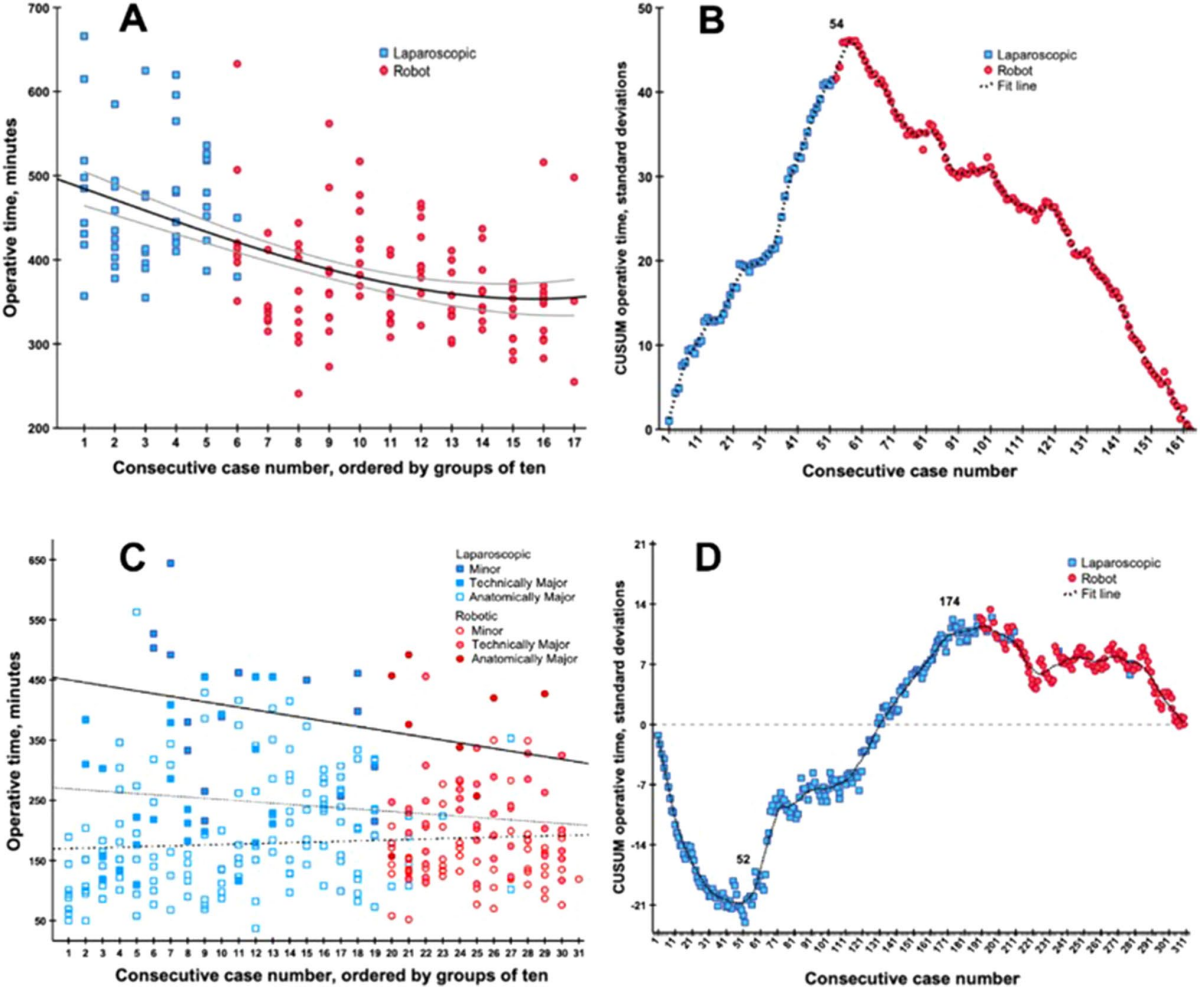
Competency learning curve of operative time in minimally invasive pancreatoduodenectomy and liver resections. **A & C** The X-axis indicated groups of 10 consecutive cases, color indicated per approach (laproscopic = blue squares, robotics = red dots), and the Y-axis indicates the combined operative time expressed in minutes (for liver resection, this way adjusted for extend of resection). The fit line indicated the mean operative time during the maturation of experience with grey lines indication in 95% confidence interval. **B & D** The X-axis indicates consecutive cases, color indicated per approach (laproscopic = blue squares, robotics = red dots) and the Y-axis indicates the CUSUM operative time expressed in standard deviations. In pancreatoduodenectomy, the label (*n* = 54) indicated the top turning point of the learning curve, hereafter, the learning curve follows a downward slope. In liver resections, the label [10] indicates the turning point where after both technically major- and anatomically major liver resections were performed. Hereafter, the label [174] indicates the top turning point of the learning curve for liver resections overall

Outcomes are shown in Table 3. The conversion rate was 7.7% for laparoscopic and 2.7% for robotic pancreatoduodenectomy (*p* = 0.143). The median operation time was 450 [IQR 411–518] for laparoscopic and 361 min [IQR 330 – 406] for robotic pancreatoduodenectomy (*P* < 0.001), see Fig. 3. Median blood loss was 350 ml [IQR 250–500] for laparoscopic and 200 ml [IQR 100–350] for robotic pancreatoduodenectomy (*P* < 0.001). Overall, after MIS pancreatoduodenectomy, major morbidity occurred in 51.5% (*n* = 84), the rate of POPF grade B/C was 35% (*n* = 57), and in-hospital/30-day mortality 3.1%. There was no significant difference in (major) complications between both approaches except for a decreased rate of delayed gastric emptying after robotic pancreatoduodenectomy (28.8% vs 9.9%; *P* = 0.009). The lymph node retrieval in case of malignancy was higher with the robotic approach (14 [IQR 11–17] vs 11 [IQR 8–15], *P* = 0.016).

**Table 3:** Patient- and surgical outcomes on all minimally invasive pancreatic resections

Characteristics	Laparoscopic pancreatoduodenectomy *N* = 52	Robotic pancreatoduodenectomy *N* = 111	*P*	Laparoscopic distal pancreatectomy *N* = 123
Operation time, minutes, median [IQR]	450 [411–518]	361 [330–406]	** < 0.001**	251 [206–322]
Blood loss, ml, median [IQR]	350 [250–500]	200 [100–350]	** < 0.001**	200 [50–350]
Drain placement	52 (100)	111 (100)	0.581	108 (87.8)
Pancreatic texture, soft, *n* (%)	35 (67.3)	70 (63.1)	0.869	65 (52.8)
Duct size in mm, median [IQR]	3 [2–4]	3 [2–5]	0.475	1 [1–2]
Vascular resection *n* (%)	2 (3.8)	3 (2.7)	0.693	1 (0.8)
Multi-visceral resection, *n* (%)	1 (1.9)	–	0.143	23 (18.7)
Conversion, *n* (%)	4 (7.7)	3 (2.7)	0.143	8 (6.5)
*Complications*				
Postoperative pancreatic fistula grade B/C	17 (32.7)	50 (45.0)	0.135	44 (35.8)
Post-pancreatectomy hemorrhage grade B/C, *n* (%)	7 (13.5)	8 (7.2)	0.198	6 (4.9)
Delayed gastric emptying grade B/C, *n* (%)	15 (28.8)	11 (9.9)	**0.009**	6 (4.9)
Bile leakage grade B/C, *n* (%)	7 (13.4)	10 (9.0)	0.386	-
Clavien Dindo > 3a, *n* (%)	22 (42.3)	62 (55.9)	0.085	36 (29.3)
Length of stay, days, median [IQR]	12 [7–19]	10 [7–21]	0.463	5 [4–7]
R0 resection in case of malignancy, *n* (%)	36 (94.7)	62 (84.9)	0.127	39 (69.6)
Reoperation, *n* (%)	4 (7.7)	14 (12.6)	0.350	2 (1.6)
Readmission, *n* (%)	6 (11.5)	26 (23.4)	0.075	31 (25.2)
In-hospital/30-day mortality, n (%)	2 (3.8)	3 (2.7)	0.693	2 (1.6)
Adjuvant therapy in patients with cancer, *n* (%)	11 (27.5)	23 (20.7)	0.949	19 (15.4)

Bold values indicate statistical significance (*p* < 0.05)

Values in parentheses are percentages unless mentioned otherwise

*IQR* inter quartile range

A total of 18 reoperations was performed in all pancreatoduodenectomies (laparoscopic *n* = 4 and robotic *n* = 14). Reasons for re-operation consisted of anastomotic leakage (*n* = 7), bleeding without endovascular options (*n* = 6), evacuation of a hematoma after bleeding (*n* = 3), bowel ischemia (*n* = 1) and trocar site herniation (*n* = 1).

The mean operative time was 402 min. Naturally, there was a gradual decrease of operative during the maturation of experience (Rho = -0.542, *P* < 0.001). For laparoscopic pancreatoduodenectomy, the mean operative time was 492 min in the first 10 procedures and 472 min in the last 10 procedures. Hereafter, in the first 10 robotic pancreatoduodenectomies, the mean operative time was 442 min and 355 min in the last 10 procedures. Overall, operative time did reach a stable plateau of 360 min. See Fig. 3A for more details. CUSUM analysis on all pancreatoduodenectomy, identified a turning point of 54 procedures, whereafter the operative time was markedly shorter than during the first 54 procedures (450 [412–513] min vs 360 [330–403] min, *P* < 0.001). The laparoscopic procedures alone did not reach a CUSUM analysis turning point. In the robotic procedures alone, there was a turning point after 7 procedures. See Fig. 3B for more details. Mortality and major morbidity did not demonstrate a significant decrease (Rho = -0.070, *P* = 0.374; Rho = 0.116, *P* = 0.142, respectively). We found no significant changes in vascular resection rates, multi-visceral resection rates, lesion size, or length of stay.

#### MIS distal pancreatectomy

In total, 123 procedures were performed laparoscopically with a median patient age of 66 years.

[IQR 54–73] and 73.2% ASA 1–2 (*n* = 90). The median annual volume of laparoscopic distal pancreatectomy was 17 [IQR 9 – 20]. The annual rate of MIS distal pancreatectomy remained stable over the years from 76% to 76.6% (*P* = 0.953). The median operation time was 251 min [IQR 206 – 322] and the median blood loss 200 ml [IQR 50–350]. Major morbidity was 29.3% with an in-hospital/30-day mortality 1.6% (*n* = 2). The rate of POPF grade B/C was 35.8% (*n* = 44). Robotic distal pancreatectomy was implemented only recently and therefore not included in this analysis.

### MIS liver resection (all procedures)

A total of 309 patients underwent MIS liver resection including 198 laparoscopic and 111 robotic procedures. As of December 2018, the MIS liver resection program became nearly completely robotic. The median annual volume of MIS liver resection during the study period was 24 resections [IQR 18–33 resections], with 50 robotic MIS liver resections in 2021. The annual implementation rate of MIS liver resection increased from 10% in 2010 to 47% in 2021 (*P* < 0.001), Supplementary Fig. 2. Since the application of the robotic approach, the use of robotics within the MIS liver resection group increased from 75% in 2019 to 96% in 2021 (*P* = 0.005).

Baseline characteristics are shown in Table 4. In the robotic group, there were more patients with ASA 3–4 (19.7% vs. 34.5%; *P* = 0.004), more patients received neo-adjuvant chemotherapy (13.1% vs 31.5%; *P* < 0.001), and more patients had previous extrahepatic abdominal surgery (30.8% vs 58.6%; *P* < 0.001). In addition, 156 minor (78.8%), 26 technically major (13.1%) and 16 anatomically major (8.1%) liver resections were performed in the laparoscopic group, compared to 61 minor (55.0%), 42 technically major (37.8%) and 8 anatomically major (7.2%) resections in the robotic group. The 2 other anatomically major liver resections were anatomical resections of 3 contiguous segments.

**Table 4:** Patient, tumor and procedural characteristics of all minimally invasive liver resections

Characteristics	MIS liver resections *N* = 309	Laparoscopic *N* = 198	Robotic *N* = 111	P
Age, years, median [IQR]	61 (48–70)	61 (47–69)	61 (50–71)	0.227
Male, *n* (%)	174 (56.3)	114 (57.6)	60 (54.1)	0.549
*ASA score*				**0.004**
ASA 1–2	231 (75.0)	159 (80.3)	72 (65.5)	
ASA 3–4	77 (25.0)	39 (19.7)	38 (34.5)	
BMI, kg/m2, median [IQR]	25.5 (23.0–29.4)	25.4 (22.9–29.3)	25.7 (23.1–29.6)	0.854
Neoadjuvant chemotherapy, n (%)	61 (19.7)	26 (13.1)	35 (31.5)	** < 0.001**
Cirrhosis (%)	30 (9.7)	23 (11.6)	7 (6.3)	0.130
Previous extrahepatic abdominal surgery (%)	126 (40.8)	61 (30.8)	65 (58.6)	** < 0.001**
Previous liver surgery (%)	25 (8.1)	17 (8.6)	8 (7.2)	0.670
**Tumor characteristics**				
*Histological diagnosis*				**0.014**
CRLM (%)	144 (46.6)	85 (42.9)	59 (53.2)	
HCC (%)	49 (15.9)	37 (18.7)	12 (10.8)	
Cholangiocarcinoma (%)	10 (3.2)	7 (3.5)	3 (2.7)	
Gallbladder carcinoma	5 (1.6)	0	5 (4.5)	
Non-CRLM (%)	11 (3.6)	7 (3.5)	4 (3.6)	
Benign (%)	90 (29.1)	62 (31.3)	28 (25.2)	
Number of lesions, median (IQR)	1 (1–2)	1 (1–2)	1 (1–2)	0.087
Size of largest lesion, mm, median (IQR)	30 (18–48)	27 (18–52)	31 (15–41)	0.531
**Procedure characteristics**				
*Type of resection*				** < 0.001**
Minor (%)	217 (70.2)	156 (78.8)	61 (55.0)	
Technically major	68 (22.0)	26 (13.1)	42 (37.8)	
Anatomically major (%)	24 (7.8)	16 (8.1)	8 (7.2)	
*Extent of resection*				** < 0.001**
*Anterior/left lateral segments (2,3,4b,5,6)*				
Wedge (%)	113 (36.6)	76 (38.4)	37 (33.3)	
Segmentectomy (%)	59 (19.1)	53 (26.8)	6 (5.4)	
Bisegmentectomy (%)	45 (14.6)	27 (13.6)	18 (16.2)	
*Posterior/superior segments (4a,7,8,1)*				
Wedge (%)	46 (14.9)	19 (9.6)	27 (24.3)	
Segmentectomy (%)	11 (3.6)	2 (1.0)	9 (8.1)	
Bisegmentectomy (%)	11 (3.6)	5 (2.5)	6 (5.4)	
*Anatomically Major*				
Left hemihepatectomy (%)	8 (2.6)	3 (1.5)	5 (4.5)	
Right hemihepatectomy (%)	13 (4.2)	10 (5.1)	3 (2.7)	
Extended left hemihepatectomy (%)	1 (0.3)	1 (0.5)	0	
Other anatomically major	2 (0.6)	2 (1.0)	0	

Bold values indicate statistical significance (*p* < 0.05)

Values in parentheses are percentages unless mentioned otherwise. Percentages may not add up due to rounding and missing data

*MILS* minimally invasive liver surgery, *IQR* inter quartile range, *BMI* body mass index, *ASA* American Society of Anaesthesiology, *CRLM* colorectal liver metastasis, *HCC* hepatocellular carcinoma

Table 5 shows the operative outcomes of all MIS liver resections stratified for approach. Overall, the median blood loss was 200 mL [IQR 50–400] with a conversion rate of 3.6%. The rate of severe complications was 10.4% and median hospital stay 4 days [IQR 2–5]. The rate of 30-day/in-hospital mortality was 1.3% (*n* = 4). Reoperations occurred in 9 patients (3.7%) and indications for reoperation included anastomotic leakage after simultaneous colorectal resection (*n* = 4), intra-abdominal bleeding (*n* = 4) and a strangulated inguinal hernia (*n* = 1). Comparing outcomes before and after introduction of the robotic approach, blood loss decreased (288 ml vs. 100 ml; *P* < 0.001) and hospital stay shortened (4 vs 3 days; *P* < 0.001) as compared to the laparoscopic approach, whereas the 30-day/in-hospital mortality did not differ significantly between both approaches (1.0% vs. 1.8%; *P* = 0.555).

**Table 5:** Operative outcomes of all minimally invasive liver resections

Characteristics	Total MILS *N* = 309	Laparoscopic liver resection *N* = 198	Robotic liver resection *N* = 111	*P*
Operation time, minutes, median [IQR]	165 (120–247)	154 (102–270)	171 (132–242)	0.236
Blood loss, mL, median [IQR]	200 (50–400)	288 (100–700)	100 (48–200)	** < 0.001**
Conversion	11 (3.6)	7 (3.5)	4 (3.6)	0.975
Complications	61 (19.7)	49 (24.7)	12 (10.8)	**0.003**
Clavien Dindo > 3a	32 (10.4)	23 (11.6)	9 (8.1)	0.332
Length of stay, days, median, [IQR]	4 (2–5)	4 (3–6)	3 (2–4)	** < 0.001**
Reoperation within 30 days	9 (3.7)	7 (5.4)	2 (1.8)	0.091
Readmission within 30 days	8 (3.3)	6 (4.5)	2 (1.8)	0.241
R0 resection in case of malignancy, *n* (%)	198 (90.4)	127 (93.4)	71 (85.5)	0.056
In hospital/30-day mortality, *n* (%)	4 (1.3)	2 (1.0)	2 (1.8)	0.555

Bold values indicate statistical significance (*p* < 0.05)

Values in parentheses are percentages unless mentioned otherwise

*IQR* inter quartile range

Additionally, Supplementary Table 1 shows the operative outcomes stratified for minor, technically major and anatomically major laparoscopic and robotic MIS liver resection. Median blood loss was less with the robotic approach in both minor (250 mL vs 50 mL; *P* < 0.001) and technically major (300 mL vs 150 mL; *P* = 0.001) resections, whilst there was no difference in the anatomically major group. Complications occurred less often with the robotic approach after both minor (17.9% vs 4.9%; *P* = 0.014) and technically major (42.3% vs. 16.7%; *P* = 0.020) MIS liver resections, as compared to the laparoscopic approach. Hospital stay was shorter with the robotic approach for both minor (4 vs. 3 days; *P* < 0.001) and technically major (6 vs. 3 days; *P* < 0.001) MIS liver resections. Operation time, conversion rates and 30-day/in-hospital mortality did not differ between both approaches in all subgroups.

For MIS liver resection, mean operative time was 209 min. Naturally, there was a gradual decrease of operative during the maturation of experience (Rho = -0.162, *P* < 0.001). For laparoscopic liver resections, the mean operative time was 96 min in the first 10 procedures and 186 min in the last 10 laparoscopic liver resection. In the first 10 robotic liver resections, the mean operative time was 194 min and 182 min in the last 10 RPD procedures. Both the mean operative time and the decrease in operative time differed between minor liver resection, technically major liver resection and anatomically major resection: 181(± 95) min, Rho = 0.121; 235(± 93), Rho -0.116; and 384(± 117), Rho -0.271, *P* < 0.001, respectively. See Fig. 3C for more details. CUSUM analysis identified a turning point of 52 liver resections, whereafter both technically major- and anatomically major liver resections were performed. The turning point of 174 liver resections indicated that, hereafter, the operative time was markedly shorter compared to the operative times of liver resections before the turning point (consecutive 52 to 174 procedures): 229 [146–333] min vs 179 [130–240] min, *P* = 0.003). The laparoscopic liver resections alone did not reach a CUSUM analysis turning point. In the robotic cases alone, there was a turning point after 86 procedures. See Fig. 3B for more details. Mortality did not demonstrate a significant decrease (Rho = -0.100, *P* = 0.079. However, major morbidity did demonstrate a significant decrease (Rho = -0.144, *P* = 0.011) CUSUM analysis revealed a turning point after 128 cases, the difference in mortality rate before and this tuning point was an odds ratio of 0.669, *P* = 0.280 (12.6% vs 8.8%).

## Discussion

This first single-center study to describe the transition from laparoscopic to robotic surgery in 600 MIS-HPB resections found the latter approach to be associated with increased implementation up to nearly half of all pancreatic and liver resections. Furthermore, the introduction of the robotic approach was associated with improved outcomes in these selected patients and low mortality. Ultimately, randomized studies will have to confirm the benefits of the robotic approach.

Large single-center studies assessing the implementation of MIS-HPB resections including both laparoscopic and robotic pancreatic and liver resections are lacking. For MIS-HPB surgery in general, one single center study reported on the stepwise implementation of 77 robotic liver resections and 68 robotic pancreatoduodenectomies, but did not include laparoscopic resections [34]. Most other single center implementation studies focused on either pancreas or liver resections and, similarly, either laparoscopic or robot surgery. The largest series on the introduction of robotic pancreatoduodenectomy (*n* = 500) comes from Pittsburgh and reported a plateau for operating time after 240 procedures of 390 min [9]. This series compares favorably with a median operating time of 360 min but this is most likely explained by the LAELAPS-3 training program wherein the Dutch surgeons were trained by the Pittsburgh team [8]. Recently, for MIS liver resection, a single center and single-surgeon retrospective Belgium study assessed the transition from 120 laparoscopic to 71 robotic liver resections [35]. The authors concluded that experience with laparoscopic MIS seems to overcome the learning curve of robotic MIS as early outcomes of robotic MIS were similar to the mastery phase of laparoscopic MIS. According to a systematic review by Müller et al., the proficiency learning curve (major complication) has not been reported for robotic pancreatoduodenectomy and may extend 25 to 80 laparoscopic pancreatoduodenectomies [36]. In this study major morbidity and mortality rates did not decrease significantly with the maturation of experience, however, it could be a result of the limited power and expanding the indication to patients with higher risks for complications. For example, mortality of pancreatoduodenectomy decreased from 8.6% to 0% after 58 cases in this study. According to a systematic review by Chua et al., the learning curve for minimally invasive hepatectomy is 50 procedures for laparoscopic- and 25 for robotic procedures [37]. Conversely, Krenzien et al., demonstrated that the learning phase for MILS adjusting for complexity is about 4 times longer, *i.e.,* 117 LLR and 93 RLR [38]. This resonates with the findings in the current study where we could adjust the CUSUM analysis of liver resections for the extent of the resection (minor, technically major, anatomically major). As complexity has not been adequately represented in learning curve models, the current study provides a nuance to studies looking at either minor or major resection only. The learning curve for major complications was 128 cases, however without a significant impact before and after the turning point.

The introduction of robotic pancreatoduodenectomy in this study was associated with a reduction in operation time and blood loss. This reduction could also be explained by increasing experience, yet Fig. 3 suggests that the reduction extends beyond the previously observed trend and is quite ‘steep’. Previous studies reporting on a shift from laparoscopic to robotic pancreatoduodenectomy are lacking. A systematic review found robotic pancreatoduodenectomy associated with a shorter hospital stay, less blood loss, and less conversion, as compared to laparoscopic pancreatoduodenectomy [39]. Notably, randomized trials focusing on robotic pancreatoduodenectomy are lacking. The recent Miami guidelines advice a minimum center volume of 20 MIS pancreatoduodenectomies per year [7]. These criteria were met in each year in our center after implementation of robotic pancreatoduodenectomy with a median of 34 procedures per year which should be taken into account when interpreting these findings.

The use of MIS liver surgery increased during the study period from 10 to 47% per year. The earlier mentioned Belgium single-center study reported an increase in the use of MIS liver resection from 45% in 2012 to 79% in 2020 [35]. Although substantially higher than the annual rates in the present series, this might be related to the referral practice for hilar cholangiocarcinoma in our center [40]. Furthermore, a single high-volume experience from Singapore reported increased use of MIS liver resections from 3.3% to 44.1% which is highly similar to the 47% in the present series [41].

The overall rate of POPF after MIS pancreatoduodenectomy in the current study (41.1%) is higher than previously reported, whereas the mortality rate (3.1%) is comparable to other retrospective studies describing first experiences with robotic and laparoscopic pancreatoduodenectomy (1.7% vs 1.4% and 4.0%) [9, 42, 43]. The high rate of POPF may be partially explained by the proactive drainage approach as was implemented in the nationwide randomized PORSCH trial which led to a 50% reduction of mortality after pancreatoduodenectomy [44]. Clearly, also a learning curve effect cannot be excluded. Only six patients used neoadjuvant therapy in this study. This is explained by the use of MIS pancreatoduodenectomy only in patients without vascular contact in the study period wherein neoadjuvant therapy was only used in patient with borderline and resectable disease.

Regarding MIS liver resection, the recent Southampton guidelines advise a stepwise implementation [14]. Several studies showed that conversion may worsen blood loss, prolong operation time, increase hospital stay, morbidity rates, and even mortality [45–47]. The conversion rate in the current study for all MIS liver resections (3.6%) was favorable and in line with previous large retrospective cohort studies [19, 46, 48, 49]. In addition, acceptable rates of major morbidity and mortality after laparoscopic and robotic MIS liver resection were observed which are in line with previous reports [19, 46, 49–51]. Randomized studies comparing laparoscopic and robotic MIS liver resection are lacking and are warranted to explore the exact value of robotic MIS liver resection in the current MIS liver resection practice.

The current findings may reflect or forecast the use of MIS-HPB resections in high-volume HPB centers worldwide. This has important implications for training of surgeons and operating staff in HPB units worldwide, including the availability of robotic systems [10, 52]. For instance, in our center we have only recently obtained a third day per week on one of the Xi surgical robots. This will allow us to now further expand the MIS-HPB program to include robotic distal pancreatectomy. It is expected that many, if not most, HPB units currently struggle to obtain sufficient robotic operating time or have no access to robotic systems at all. Future studies are needed to assess whether the higher operative costs with robotic surgery are compensated by improved outcome and shorter hospital stay. Although the costs of robotic surgery are expected to decrease with the arrival of new robotic systems, ultimately randomized studies will have to confirm the benefit of robotic as compared to laparoscopic and open MIS-HPB surgery.

The current study has several limitations which should be taken into account. First, procedures were performed in selected patients. However, the selection criteria are provided in the methods section and aim to safeguard good patient outcome. Second, this study was performed in a high volume setting by a small group of specifically trained, and procedure dedicated surgeons. One surgeon was present during all laparoscopic and robotic pancreatoduodenectomy procedures and another surgeon during all robotic liver resections. This was done by design in order to minimize negative learning curve effects. As a result, the impact of surgeon training and experience could not be assessed and the results may not be generalizable to centers who disperse MIS-HPB resections over a larger group of surgeons. However, all surgeons performing minimally invasive pancreatic surgery had participated in the LAELAPS-1, -2, and -3 training programs [8, 23, 24]. Third, the outcomes of robotic and laparoscopic MIS-HPB resections cannot be compared directly because of the ongoing learning curve. It may be that with ongoing experience outcomes in most recent years would also have improved. This cannot be excluded, but when assessing operating times for MIS pancreatoduodenectomy (Fig. 3) there seems to be a rather steep improvement with the introduction of robotic surgery. This also the reasons that no (propensity-score) matching was performed. Fourth, the overall generalizability of our findings might be hampered by the use of specific training programs in our center (i.e., LAELAPS 1–2-3). However, the LEARNBOT training program for robot pancreatoduodenectomy is currently ongoing in Europe for high volume centers (www.e-mips.com/learnbot) and soon the LIVEROBOT robot liver training program will start. Both are endorsed by E-AHPBA and provide European HPB units which aim to start a robot MIS-HPB program the option to follow designated training programs.

A major strength of this study is that it demonstrates the implementation and outcome of robotic surgery in a large cohort of 600 MIS-HPB pancreatic and liver resections using a structured approach to patient selection and surgical training.

In conclusion, the implementation of robotic surgery in MIS-HPB appeared to be safe and feasible in a high-volume center leading to an implementation approaching half of all pancreatic and liver resections. Adequate training, a high-volume practice, and a deliberate team-based approach to these procedures may be beneficial. Ultimately, randomized studies will have to confirm the benefits of MIS-HPB resections including robot-assisted surgery.

## Supplementary Information

Below is the link to the electronic supplementary material.

Supplementary file1 (TIFF 12924 KB)

Supplementary file2 (TIFF 12924 KB)

Supplementary file3 (JPG 41 KB)

Supplementary file4 (DOCX 16 KB)
