# Correction: Impact of shifting from laparoscopic to robotic surgery during 600 minimally invasive pancreatic and liver resections

**DOI:** 10.1007/s00464-022-09848-w

**Published:** 2022-12-27

**Authors:** Anouk. M. L. H. Emmen, B. Görgec, M. J. W. Zwart, F. Daams, J. Erdmann, S. Festen, D. J. Gouma, T. M. van Gulik, J. van Hilst, G. Kazemier, S. Lof, S. I. Sussenbach, P. J. Tanis, B. M. Zonderhuis, O. R. Busch, R. J. Swijnenburg, M. G. Besselink, for HPB-Amsterdam

**Affiliations:** 1 Department of Surgery, Amsterdam UMC, University of Amsterdam, Amsterdam, The Netherlands; 2 Cancer Center Amsterdam, Amsterdam, The Netherlands; 3 Department of Surgery, Amsterdam UMC, Vrije Universiteit, Amsterdam, The Netherlands; 4 Department of Surgery, OLVG, Amsterdam, The Netherlands

## Abstract

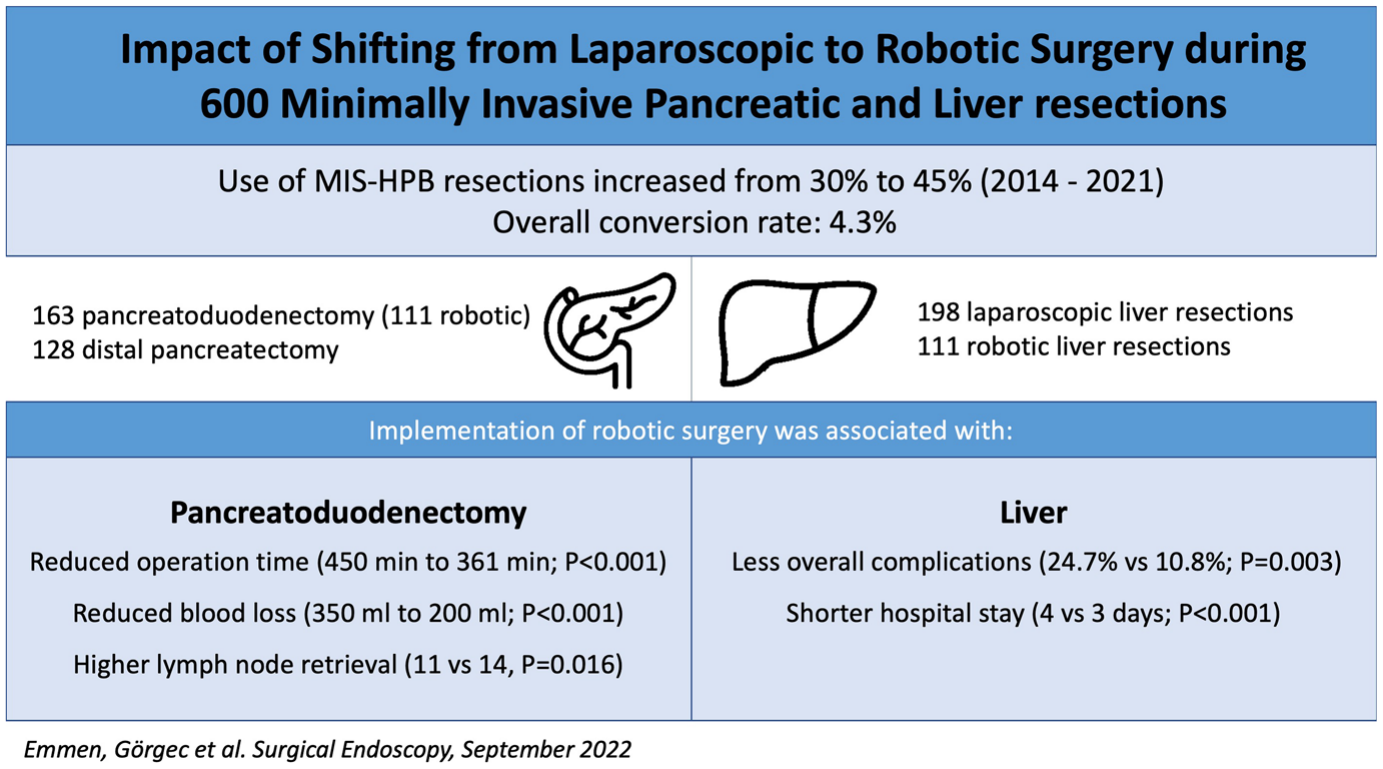

**Correction to: Surgical Endoscopy** 10.1007/s00464-022-09735-4

The original online version of this article was revised to correct a typo in the presentation of “for HPB-Amsterdam” in the contributors' list and to correct an error in the graphical abstract. The original article has been corrected.

