# Supplementary figures and images for: Impact of shifting from laparoscopic to robotic surgery during 600 minimally invasive pancreatic and liver resections
Source: Surg Endosc. 2022 Nov 18;37(4):2659–72. doi: 10.1007/s00464-022-09735-4 (PMC10082117; doi:10.1007/s00464-022-09735-4)

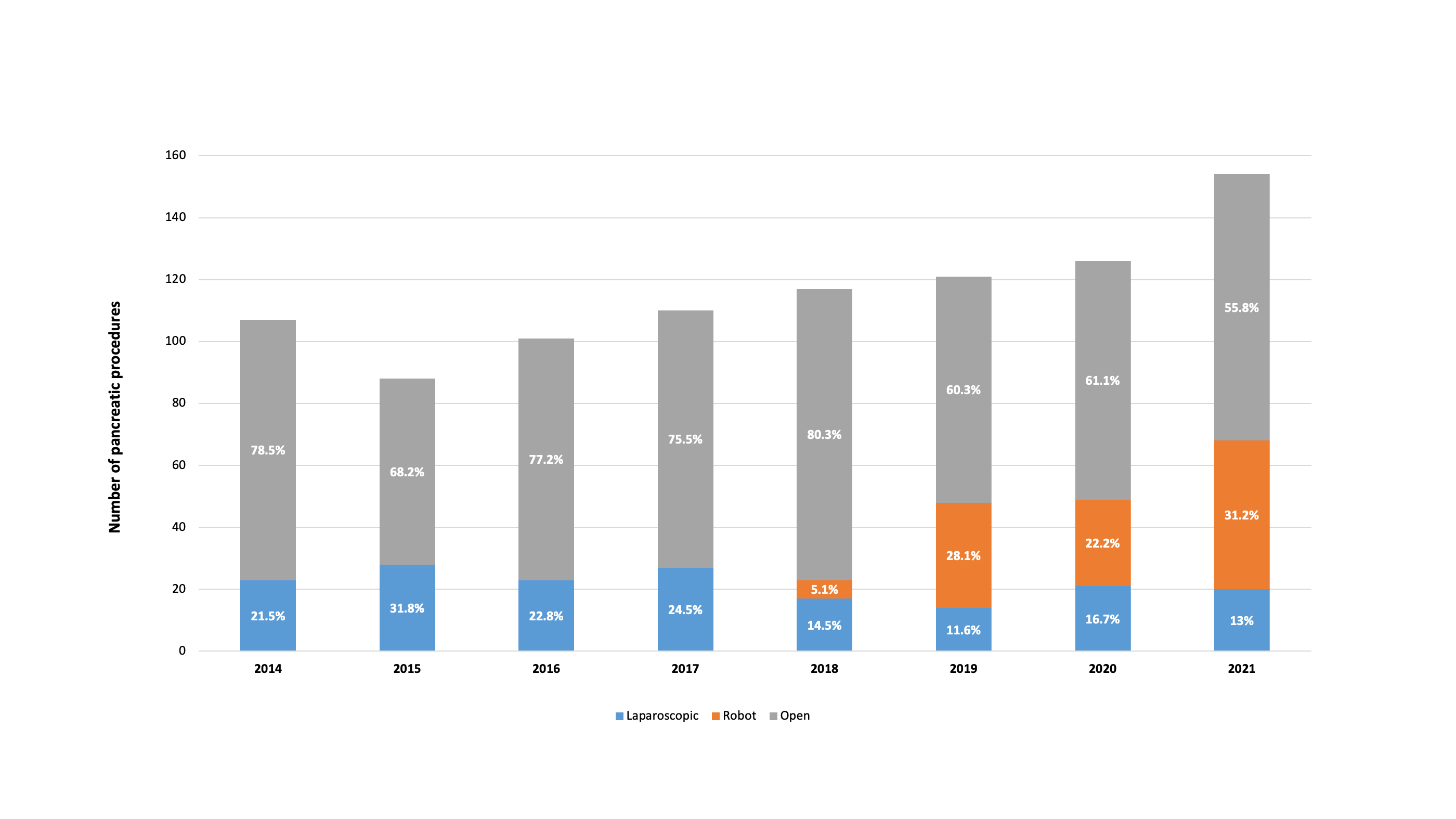

Supplement: Supplementary file 1 — Supplementary file1 (TIFF 12924 KB) [file 464_2022_9735_MOESM1_ESM.tiff]

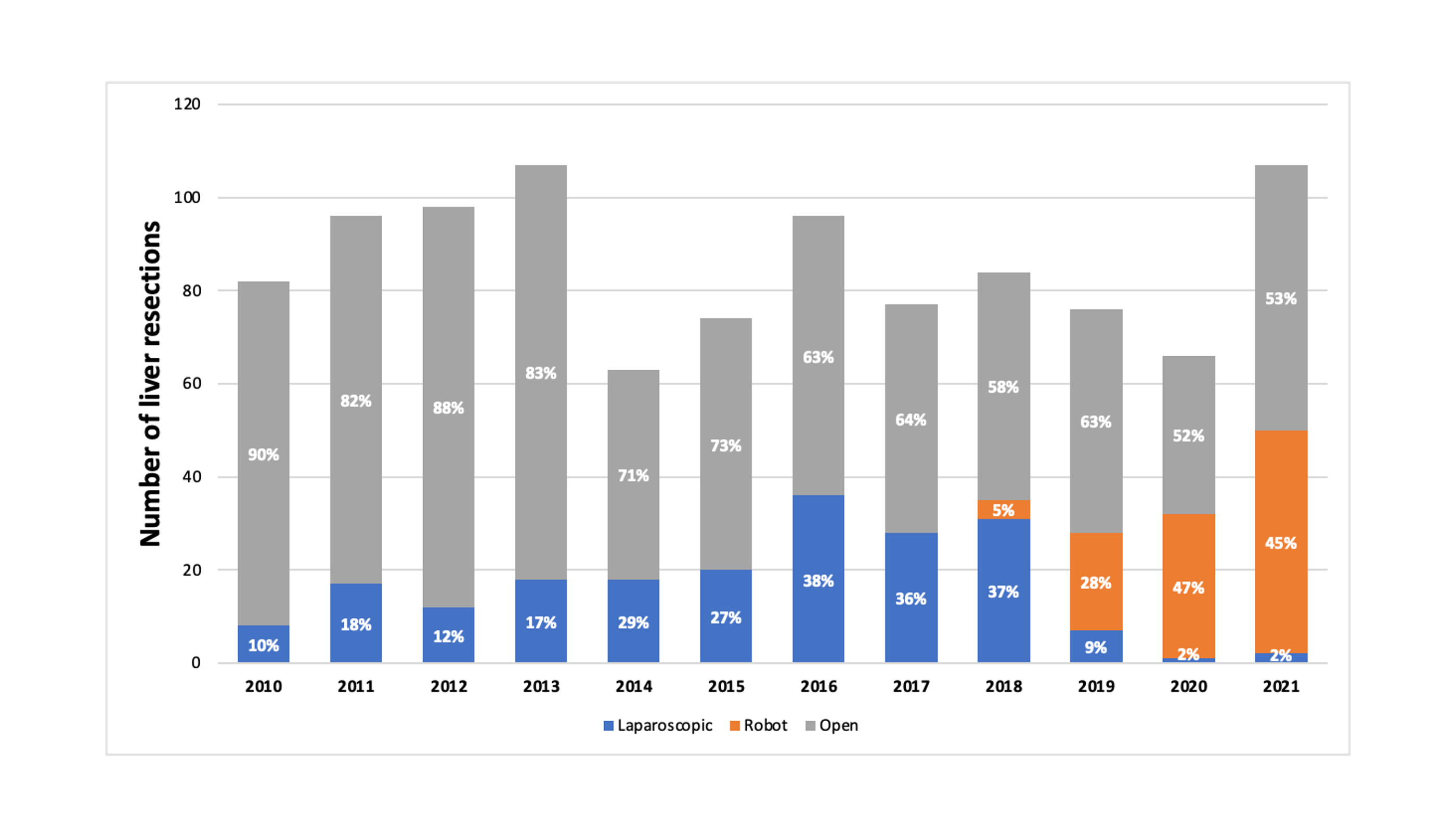

Supplement: Supplementary file 2 — Supplementary file2 (TIFF 12924 KB) [file 464_2022_9735_MOESM2_ESM.tiff]

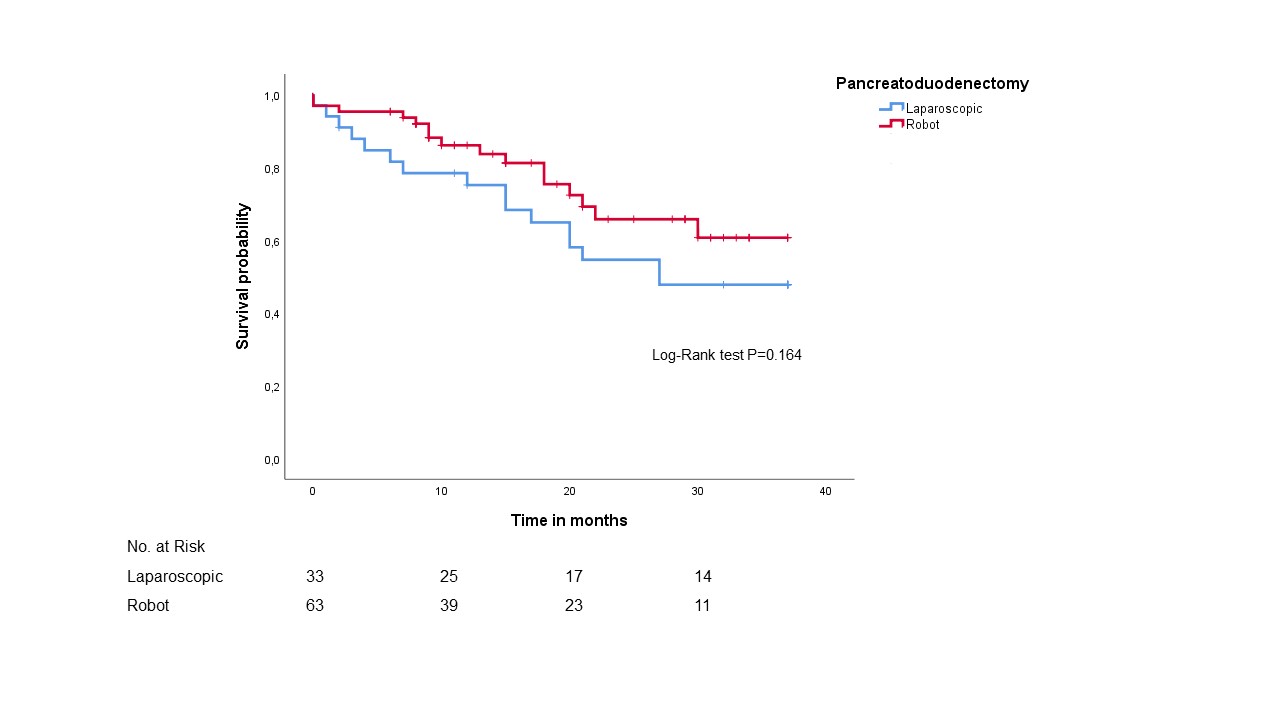

Supplement: Supplementary file 3 — Supplementary file3 (JPG 41 KB) [file 464_2022_9735_MOESM3_ESM.jpg]
