# Supplementary material for: Impact of shifting from laparoscopic to robotic surgery during 600 minimally invasive pancreatic and liver resections
Source: Surg Endosc. 2022 Nov 18;37(4):2659–72. doi: 10.1007/s00464-022-09735-4 (PMC10082117; doi:10.1007/s00464-022-09735-4)
Supplement: Supplementary file 4 — Supplementary file4 (DOCX 16 KB) [file 464_2022_9735_MOESM4_ESM.docx]

| **TABLE 6. Operative outcomes stratified for minor, technically major and anatomically major laparoscopic and robotic liver resection** | | | | | | | | | |
| --- | --- | --- | --- | --- | --- | --- | --- | --- | --- |
|  | **Minor** | |  | **Technically major** | |  | **Anatomically major** | |  |
| **Characteristics** | **Laparoscopic**  **N=156** | **Robotic**  **N=61** | **P** | **Laparoscopic**  **N=26** | **Robot**  **N=42** | **P** | **Laparoscopic**  **N=16** | **Robot**  **N=8** | **P** |
| Operation time, minutes, median [IQR] | 140 (95-186) | 141 (122-186) | 0.158 | 222 (166-345) | 208 (157-270) | 0.315 | 436 (282-521) | 376 (257-457) | 0.336 |
| Blood loss, mL, median [IQR] | 250 (100-600) | 50 (20-100) | **<0.001** | 300 (200-700) | 150 (50-300) | **0.001** | 500 (213-788) | 435 (138-500) | 0.452 |
| Conversion | 5 (3.2) | 2 (3.3) | 0.978 | 2 (7.7) | 1 (2.4) | 0.300 | 0 | 1 (12.5) | 0.149 |
| Complications | 28 (17.9) | 3 (4.9) | **0.014** | 11 (42.3) | 7 (16.7) | **0.020** | 10 (62.5) | 2 (25.0) | 0.083 |
| Severe complications | 14 (9.0) | 1 (1.6) | 0.056 | 3 (11.5) | 6 (14.3) | 0.745 | 6 (37.5) | 2 (25.0) | 0.540 |
| Length of stay (days), median, [IQR] | 4 (3-5) | 3 (2-4) | **<0.001** | 6 (4-8) | 3 (2-4) | **<0.001** | 8 (5-11) | 5 (4 -11) | 0.383 |
| Reoperation within 30 days | 5 (5.5) | 0 | 0.063 | 2 (7.7) | 2 (4.8) | 0.618 | 1 (7.7) | 0 | 0.421 |
| Readmission within 30 days | 4 (4.1) | 1 (1.6) | 0.385 | 1 (3.8) | 1 (2.4) | 0.728 | 1 (9.1) | 0 | 0.381 |
| R0 resection, in case of malignancy,  n (%) | 98 (94.2) | 39 (88.6) | 0.236 | 21 (100) | 30 (88.2) | 0.103 | 8 (72.7) | 2 (40.0) | 0.210 |
| 30 day / in hospital mortality, n (%) | 0 | 0 | 1 | 1 (3.8) | 2 (4.8) | 0.858 | 1 (6.3) | 0 | 0.470 |
| *Values in parentheses are percentages unless mentioned otherwise. RLS = Robotic Liver Surgery; IQR = inter quartile range* | | | | | | | | | |
